# Clinical performance of a glass-hybrid system in comparison with a resin composite in two-surface class II restorations: a 5-year randomised multi-centre study

**DOI:** 10.1007/s00784-024-05491-y

**Published:** 2024-01-20

**Authors:** Ivana Miletić, Anja Baraba, Silvana Jukić Krmek, Tamara Perić, Dejan Marković, Matteo Basso, Cigdem Atalayin Ozkaya, Hande Kemaloglu, Lezize Sebnem Turkun

**Affiliations:** 1 Department of Endodontics and Restorative Dentistry, School of Dental Medicine, University of Zagreb, Gunduliceva 5, 10 000 Zagreb, Croatia; 2 Clinic for Pediatric and Preventive Dentistry, School of Dental Medicine, University of Belgrade, Dr Subotica 11, Belgrade, 11000 Serbia; 3 Center of Minimally Invasive, Aesthetic and Digital Oral Rehabilitation (CROMED), IRCCS Galeazzi Orthopaedic Institute, University of Milan, Via Riccardo Galeazzi 4, 20161 Milan, Italy; 4 Department of Restorative Dentistry, Ege University School of Dentistry, Erzene Mah. Ankara Cad. No:172/109, 35040 Bornova/Izmir, Turkey

**Keywords:** Glass-hybrid system, Clinical study, Nano-hybrid resin composite, Two-surface restoration

## Abstract

**Objective:**

To evaluate the 5-year clinical performance of a glass hybrid restorative system and a nano-hybrid resin composite in moderate to large two-surface class II cavities.

**Materials and methods:**

This study was carried out by dental schools in Zagreb, Croatia; Izmir, Turkey; Belgrade, Serbia; and Milan, Italy. A total of 180 patients requiring two class-II two-surface restorations in the molars of the same jaw were recruited. The teeth were randomly restored with either a nano-hybrid resin composite (Tetric EvoCeram, Ivoclar Vivadent) or a glass-hybrid material (EQUIA Forte, GC). During the 5-year follow-up, two calibrated evaluators at each centre scored the restorations annually using the FDI-2 scoring system. The survival rates were calculated using the Kaplan–Meier method and compared using non-parametric matched pair tests (*p* < 0.05).

**Results:**

There were no statistically significant differences between the overall survival and success rates of the two types of restorations (p>0.05). The success rates (FDI-2 scores 1–3) for EQUIA Forte were 81.9% (average annual failure rate: 3.9%) and 90.7% for Tetric EvoCeram (average annual failure rate: 1.9%). The survival rates (FDI-2 scores 1–4) for EQUIA Forte and Tetric EvoCeram were 94.5% and 94.4%, respectively, with an average annual failure rate of 1.1%.

**Conclusions:**

In terms of success and survival rates, both the glass-hybrid restorative system and the nano-hybrid resin composite have been shown to perform satisfactorily.

**Clinical relevance:**

The results of this study indicate that EQUIA Forte can be one of the therapeutic options for moderate to large two-surface class II restorations of posterior teeth.

## Introduction

Dental amalgam has been a standard material for posterior restorations for more than a century due to its ease of handling, durability [1, 2] and affordability for many patients [3]. However, due to the Minamata Convention on Mercury [4], the trend worldwide is to reduce or eliminate the use of amalgam fillings. Beyond concerns regarding the harmful effects of mercury and other components of amalgam alloys, there is an increasing preference for restorations that closely resemble natural tooth colour, which has led to increased use of resin composites, glass-ionomer cements (GICs) and their modified forms [5]. Resin composite materials (CO) are known for their physical and aesthetic properties. They have a proven longevity of 10–12 years with an annual failure rate of 2–3% [6], with physical properties similar to those of metal alloys [7]. However, multi-surface CO restorations in posterior teeth require more prolonged treatment times and precise technical skills. Glass-ionomer cements are less sensitive with regard to placement techniques but are relatively brittle due to their lower flexural strength and wear resistance [8–10]. Therefore, they have been considered traditionally as temporary or provisional restorative materials, especially for larger dental defects, where resin composites and metal alloys have proven to be reliable solutions [11]. To enhance the strength and wear resistance of conventional GICs, there have been improvements to their consistency, with the introduction of high-viscosity GICs. In addition, to protect these materials, it has been proposed to apply a coating of nano-filled resin to protect and additionally cover the surface pores and thus improve the mechanical properties of the material [12]. The clinical behaviour of this group of materials has been investigated in many different clinical studies. Clinically acceptable survival rates after follow-up periods of up to 10 years in smaller restorations have been reported [12–20].

Glass-hybrid cement (GH) has been developed in recent years. It combines ultrafine and highly reactive glass particles, evenly distributed in the structure of the glass powder, with high-molecular-weight polyacrylic acid, which is expected to result in improved mechanical properties [21]. Indeed, *in vitro* studies have shown increased flexural strength [22] and wear resistance of GH [23], which can be attributed to a multifunctional monomer layer placed over the restoration to seal defects on the material surface. As GH technology is relatively new, clinical data are still limited. Previously published 2-year results of a multi-centre clinical study found similar survival rates for the GH and the nano-hybrid resin CO at 93.6% and 94.5%, respectively, when tested in load-bearing two-surface class II restorations [24]. Similarly, comparable clinical performance was reported for the GH and the micro-filled resin hybrid CO tested in extended class II cavities over a 24-month evaluation period [25]. A survival rate of 98% was observed for GH restorations in hypo-mineralised permanent molars [26]. However, a significantly lower survival rate of GH class II restorations compared to conventional GICs, and bulk-fill resin CO was reported by Balkaya and Arslan [27]. Medium- and long-term clinical studies are still scarce, and further investigation of their clinical performance is needed.

The aim of the present split-mouth study was to compare the clinical outcomes of a GH restorative system to a nano-hybrid resin CO in moderate to large two-surface class II restorations placed in four different countries using the FDI-2 criteria. The null hypothesis tested was that no differences in the clinical performance of the glass-hybrid restorative system EQUIA Forte and the nanohybrid resin composite Tetric EvoCeram would be detected after 5 years.

## Materials and methods

### Ethical considerations

The study was approved by the ethics committees of the participating centres (School of Dental Medicine, University of Zagreb, Croatia, No: 05-PA-26-7/2015; Ege University, School of Medicine, Izmir, Turkey, No: 18-11.1T/15; Galeazzi Orthopaedic Institute, Milan, Italy, No: 117/INT/2015; and School of Dental Medicine, University of Belgrade, Serbia, No: 36/24). The clinical study was registered at clinicaltrials.gov under the number NCT02717520.

### Study design and participants

The study design has been previously published [24]. The design of this study was longitudinal (with a 5-year follow-up period), split-mouth (with equal allocation given to the left and right sides of the same jaw), prospective, multi-centre and with concealed randomised allocation to two treatment groups. It was conducted in and by four European clinical centres and dental schools (Zagreb, Croatia; Izmir, Turkey; Milan, Italy; and Belgrade, Serbia).

Patients were recruited over a 9-month period (September 14, 2015, until June 14, 2016) from patients who attended one of the university clinics and required two class II, two-surface restorations in the molar region of the same jaw. The inclusion and exclusion criteria are shown in Table 1. Before signing the consent forms, all patients were informed by the participating clinicians about the purpose of the study and the procedures and risks related to it, as well as their rights, including the right to withdraw from the study at any time.

**Table 1:** Inclusion/exclusion criteria for patients included in the study

Inclusion criteria	Individuals over 18 years old with permanent dentition. Individuals in need of two restorative treatments on vital posterior molar teeth on the same jaw (primary caries or replacement of existing restoration). Restorations limited to two surfaces having both antagonist and adjacent tooth and at least one occlusal contact. Favourable and stable occlusal relationship between the remaining teeth. Reliable, cooperative, willing to participate in the study and able to return for periodic follow-ups.
Exclusion criteria	Individuals with full dentures or crowns and bridges in occlusal contact with teeth indicated for restorative treatment. Individuals with a history of drug abuse, addiction to medication or alcohol abuse. Pulp exposure while caries excavation. Known unavailability for recall visit(s). Allergy to any product used in the study. Severe bruxism. Individuals with unstable medical or physiological conditions. Pregnant, lactating subjects or intending to become pregnant during the study.

A randomisation sequence was generated using a random number generator with uniform distribution and a range of [0–1]. All random numbers smaller than 0.5 were assigned to a glass-hybrid restoration on the left side and resin CO on the right side (group A), while the random numbers 0.5 or larger were assigned to having a glass-hybrid restoration on the right side (group B). The random sequence results were put in closed envelopes and distributed to the participating centres. After the patient was recruited and found to meet the inclusion criteria, the envelope was opened, and the patient was allocated to one of the two groups. There was no blinding beyond this point. Each participating patient received two restorations: a GH material (EQUIA Forte, GC, Tokyo, Japan) and a nano-hybrid resin CO (Tetric EvoCeram, Ivoclar Vivadent, Schaan, Liechtenstein). The characteristics of the materials are shown in Table 2. The patients’ baseline characteristics are shown in Table 3.

**Table 2:** Composition, type and manufacturer of the materials tested

Material	Type	Manufacturer	Composition
EQUIA Forte	Glass hybrid	GC (Tokyo, Japan)	Powder: 95% strontium fluoroalumino-silicate glass, 5% polyacrylic acid liquid, 40% aqueous polyacrylic acid
Tetric EvoCeram	Nano-hybrid resin composite	IvoclarVivadent (Schaan, Liechtenstein)	Dimethacrylates 16. 8% (Bis-GMA, Bis-EMA, UDMA, ethoxylated Bis-EMA), fillers 82% (barium glass, ytterbium trifluoride, mixed oxide, SiO_2_)
EQUIA Forte Coat	Low-viscosity nano-filled resin	GC (Tokyo, Japan)	40–50% methyl methacrylate 10–15% colloidal silica 0. 09% camphorquinone 30–40% urethane methacrylate 1–5% phosphoric ester monomer
AdheSE	Self-etching two-component adhesive system	IvoclarVivadent (Schaan, Liechtenstein)	AdheSE Primer: dimethacrylate, phosphonic acid acrylate, initiators and stabilisers in an aqueous solution AdheSE Bond: HEMA, dimethacrylate, silicon dioxide, initiators and stabilisers
Cavity conditioner	Surface conditioner	GC (Tokyo, Japan)	20% polyacrylic acid, 3% aluminium chloride hexahydrate, distilled water

**Table 3:** Baseline characteristics of the patients included in the study

	Total	
	N	%
	180	100.0
Gender		
Female	116	64.4
Male	64	35.6
Centre		
Croatia	60	33.3%
Italy	32	17.8%
Serbia	28	15.6%
Turkey	60	33.3%
Age (years)		
Median	27	
Interquartile range	22–39	
Range	18–77	

### Interventions: restorative procedures

As a first step, all tooth surfaces were cleaned to remove dental plaque and salivary pellicle using a fluoride-free prophylactic paste (Cleanic, Kerr, Orange, CA, USA) along with a polishing brush in a slow handpiece. Gingival bleeding was recorded, and tooth sensibility was confirmed using ethyl chloride. Local anaesthesia was administered for all cavity preparations. Digital pre-operative photographs (direct, occlusal, buccal and oral view) were taken. To ensure a dry operative field, cotton rolls and high-speed suction were employed for EQUIA Forte and a rubber dam for Tetric EvoCeram.

Teeth with existing restorations or with primary caries were prepared using high-speed spherical and cylindrical diamond burs (1.204.023 and 9120.314) (Komet, Lemgo, Germany) with water-cooling. Then, carious tissues were removed using a hand instrument and/or spherical slow-speed round burs. At the periphery of the cavity, preparation was performed to ensure the cavity margins were located in sound enamel and cavity walls in hard dentine. At the cavity floor, selective removal until firm/leathery dentine was done [28]. No selective removal to soft dentine was performed. All cavities were prepared without bevelling and were restored using a pre-contoured sectional matrix system (Palodent Plus, Dentsply, York, PA, USA).

Cavities were restored with the GH EQUIA Forte, following the manufacturer’s instructions. The smear layer was modified using 20% polyacrylic acid for 10 s (Cavity Conditioner, GC), thoroughly rinsed and briefly air-dried, ensuring not to desiccate the surface. After mixing EQUIA Forte capsules for 10 s in a capsule mixer (Silvermix90, GC), the material was packed into the cavities in bulk using a capsule applicator. Following the formation of major grooves and fissures with hand instruments and a 2.5-min initial setting time, occlusal contact was checked using two-sided coloured articulating paper (blue for occlusion and red for articulation, 20 μm thickness). Cervical adaptation and proximal contact were checked with dental floss and adjusted as required with flexible discs (952.900.140 and Compo System, Komet). After drying the restorations, a layer of EQUIA Forte Coat was applied to the occlusal surfaces and light cured for 20 s using an LED curing lamp (D-Light; GC) at 1200 mW/cm^2^.

For Tetric EvoCeram resin CO restorations, a rubber dam was placed to ensure a dry work field before cavity preparation. The enamel was selectively etched with 37% phosphoric acid, and a two-step self-etching adhesive (AdheSE, Ivoclar Vivadent) was applied to the enamel and dentin according to the manufacturer’s instructions and was light cured for 20 s (1200 mW/cm^2^) using an LED curing lamp (D-Light, GC). The material was placed in 2-mm increments, and each layer was polymerised for 20 s from the occlusal aspect. The restorations were cured from the buccal and palatal/lingual directions after removing the matrix system. The restorations were finished with a high-speed handpiece with fine and extra fine-grit flame diamond burs (H135F.314.014 and 368LEF.314.016, Komet) for gross finishing, while fine finishing was performed using carbide burs (H48LF.314.012, Komet) and a slow-speed handpiece and flexible discs (952.900.140 and Compo System, Komet). Proximal surfaces were polished using fine polishing strips. Occlusion and articulation were checked and corrected as required following the same procedure used in the glass-hybrid group. The restorations were then polished with rubber points (9523UF.204.030, Komet) and diamond polishing paste (Gradia DiaPolisher, GC).

For both materials, A2 shade was used. Both restorations were placed no longer than 1 week apart. Two operators with more than 3 years of clinical experience in conservative dentistry and instructed by the study coordinator placed all restorations in all study centres (eight operators in total). A total of 360 restorations were placed. Of 180 EQUIA Forte restorations, 50 teeth were restored due to the primary caries lesions, and 130 were replacements of defective restorations. Of 180 Tetric EvoCeram restorations, 51 restored primary caries lesions, and 129 were replacements of defective restorations. No indirect pulp capping was performed. No dental radiography was taken during and immediately after the restorative procedures or routinely at the recall periods.

### Evaluation of the restorations

The evaluators were two experienced clinicians at each site, who were calibrated for FDI-2 criteria and were blinded to the clinical procedures. After being individually calibrated on the e-calib web page, the evaluators assessed a set of 11 restoration pictures, assigning scores to each. The evaluation results showed excellent inter-rater agreement, with average values ranging from 0.939 to 0.989 for the following variables: surface staining, marginal staining, overall functional properties, material fracture and retention and marginal adaptation. For the rest of the FDI-2 criteria, there was zero disagreement between the evaluators for all tested pictures. However, it was not feasible to blind the evaluators to the restorative material used, as these had visibly distinct appearances.

**Fig. 1:**
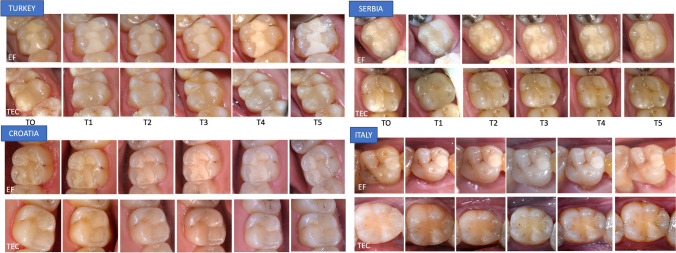
Two-surface restorations performed in different countries with EQUIA Forte (EF; upper rows) and Tetric EvoCeram (TEC; lower rows) and scored as excellent during the yearly recall periods (T0 to T5)

The patients were followed up at 1 week (baseline) and after 1, 2, 3, 4, and 5 years (Fig. 1). The restorations were evaluated according to the FDI-2 criteria [29, 30]. The criteria used for the evaluations included aesthetic aspects (only marginal and surface staining), functional characteristics (all criteria except occlusion and wear, which were measured objectively and will be reported in a separate paper) and biological considerations (all available criteria). The colour match and translucency scores were not used because they were considered clinically less important for the restorations in the posterior region [31]. Each restoration received a score on a scale from 1 to 5: 1-clinically excellent; 2-clinically good; 3-clinically satisfactory; 4-clinically unsatisfactory but reparable; and 5-poor, requiring replacement.

The primary outcomes were the survival rate and the success rate of the restorations. The survival was defined as a restoration not requiring replacement (FDI-2 scores of 1-4) and the success as a restoration not needing replacement or repair (FDI-2 scores of 1–3). The secondary outcomes of the trial were the FDI-2 properties of the two materials.

The differences between evaluators were resolved through consensus. Each restoration was documented by photographs taken from direct, occlusal, buccal and oral directions, as well as before and after restoration and at each follow-up recall.

**Fig. 2:**
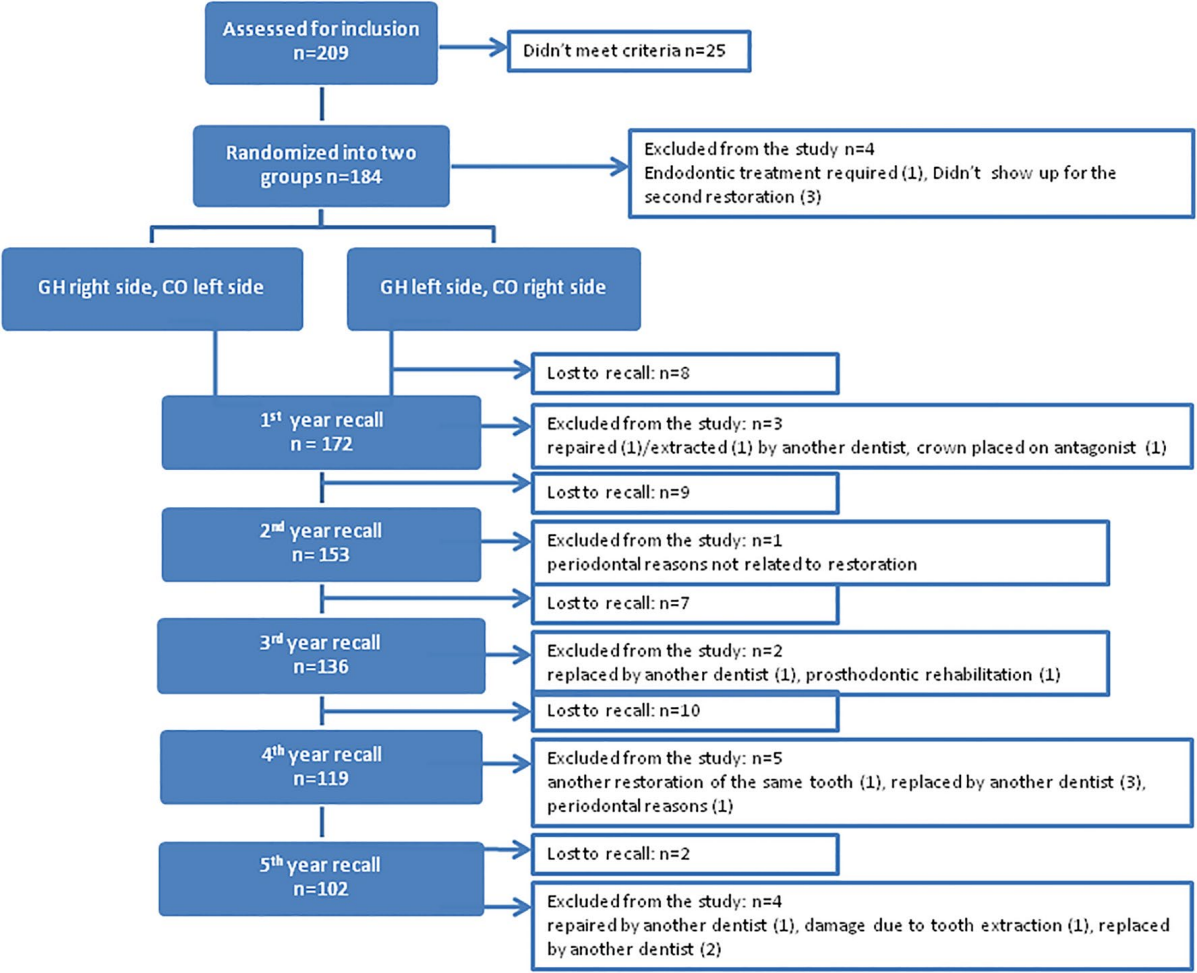
Study flow

The participants were reminded of the follow-up visit 4 times before being declared ‘not attending to follow-up’, using different methods (i.e. mobile phone, message via social media and email). The study flow is shown in Fig. 2. Out of the total of 209 patients assessed for inclusion in the study, 184 of them initially met the inclusion/exclusion criteria (Table 3). However, four of them were subsequently excluded because one patient needed endodontic treatment for the study tooth during the restorative procedure and three patients failed to show up for the restoration of the second tooth.

### Sample size, power and statistical analysis

The sample size was chosen to achieve a statistical power of 0.8 in detecting whether the tested glass-hybrid material had a restoration survival rate at least 10% lower than that of resin CO materials, with an error of 0.05. The calculation was performed using G*Power software (University of Kiel, Germany). The required sample size was 122 samples, and the final sample size was set to 180, as a 70% recall rate was expected. The dropout rate was lower than 30%, but due to the COVID-19 pandemic, the sample was further reduced by excluding some patients from the study because they received interventions from other dentists, as travel was limited at that time. A post hoc power analysis showed that the power of the study to detect differences in success rates was 0.77.

The inter-scorer agreement was tested using interclass correlation in mixed mode with the agreement criterion. Survival analysis was used for failure rates. The survival/success rates were calculated using Kaplan-Meier analysis. The equality of the Kaplan-Meier 5-year survival/success rates was tested using the exact binomial *p*-value variant of the McNemar paired samples test. The mean survival times were not statistically compared in this study: the patients were excluded after the failure of one of the restorations, and the survival times and their determinants will be published separately.

Annual failure rate was calculated according to the formula (1 − *y*)^5^ = 1 − *x*, where *x* expresses the total failure (non-success or non-survival rate) and *y* is the average annual failure rate.

The statistical significance cut-off was set at 0.05. The samples that needed no revision, repair or replacement and were followed for 5 years (*n* = 95) were evaluated by FDI-2 scores using the Wilcoxon signed-rank test. All analyses were performed in SPSS software on the Windows platform, with a *p*-level of 0.05.

### Results

The overall recall rate after 5 years for all centres was 73.9% (Table 4). Four patients could not attend their recalls during the COVID-19 pandemic period during the fourth year. However, they returned for the fifth-year control, and their restorations were re-scored. A total of 15 patients (8.3% of the original sample size, Table 4) were excluded from the study.

**Table 4:** Number of patients who were excluded, did not attend and were recalled for each year. The total recall rate was defined as a percentage

Time point	Actual respondents	Respondents excluded from the study	Respondents lost to recall	Recall rate (%)
Baseline	180	0	0	
1 year	172	3	8	95.6%
2 years	153	1	17	90.0%
3 years	136	2	24	85.0%
4 years	119	5	34	77.8%
5 years	102	4	36	73.9%

**Fig. 3:**
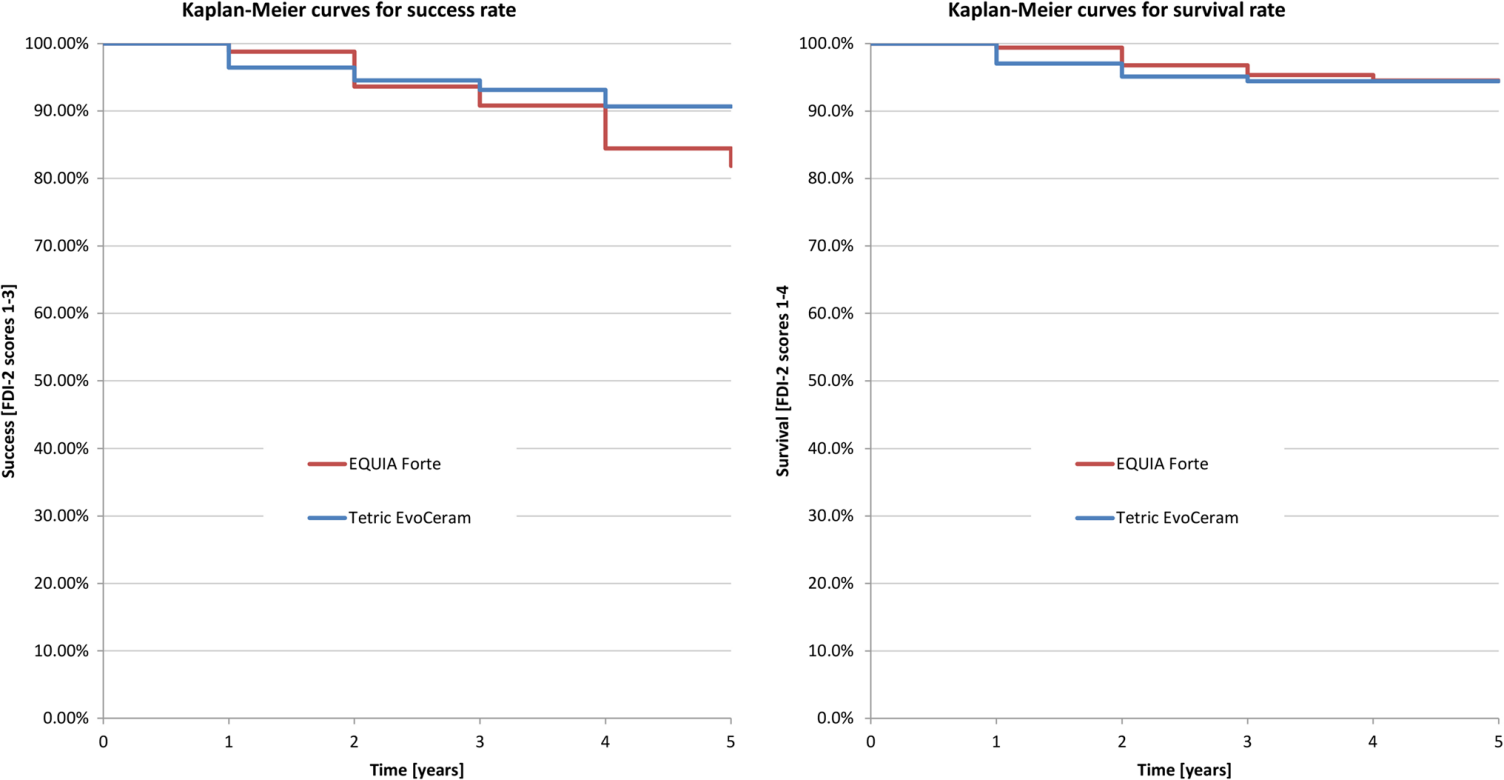
Survival and success rates of EQUIA Forte and Tetric EvoCeram restorations during the study periods

**Fig. 4:**
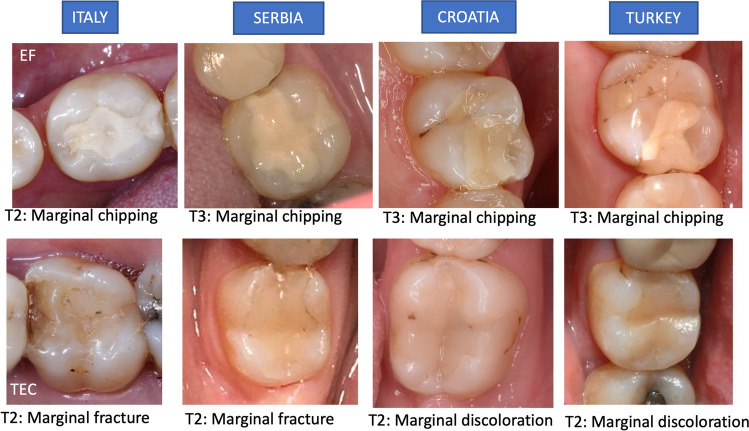
Marginal chippings and their occurrence years in EQUIA Forte (EF) restorations observed in every country (upper row). Marginal fractures and discolorations observed in Tetric EvoCeram (TEC) restorations in different time periods in every country (lower row)

No statistically significant differences (*p* > 0.05) were observed in the overall survival rates and success rates of the two types of restorative materials (Fig. 3 and Table 5). The success rates (FDI-2 scores 1–3) were 81.9% for EQUIA Forte (average annual failure rate was 3.9%) and 90.7% for Tetric EvoCeram (average annual failure rate was 1.9%). The survival rates (FDI-2 scores 1–4) were 94.5% and 94.4%, respectively, for EQUIA Forte and Tetric EvoCeram, with an average annual failure rate of 1.1% for both materials (Fig. 3 and Table 5). A total of 8 EQUIA Forte and 9 Tetric EvoCeram restorations had to be replaced. The main reasons were postoperative sensitivity and fracture of material and retention (Table 6). A total of 17 EQUIA Forte and 5 Tetric EvoCeram restorations needed repair. The main reason for restoration repair was fracture of material and retention, accounting for all 17 EQUIA Forte repairs (100%) and 3 (60%) Tetric EvoCeram repairs, while 2 (40%) repairs were due to recurrence of caries, erosion and abfraction (Fig. 4 and Table 6).

**Table 5:** Reasons for repair and replacement of the materials tested

		Reason	*N*	%
Repair	EQUIA Forte, *n* = 17	Fracture of material and retention	17	100%
	Tetric EvoCeram, *n* = 5	Fracture of material and retention	2	40%
		Recurrence of caries, erosion and abfraction	3	60%
Replacement	EQUIA Forte, *n* = 8	Postoperative sensitivity and tooth vitality	4	50%
		Fracture of material and retention	3	38%
		Recurrence of caries, erosion and abfraction	1	13%
	Tetric EvoCeram, *n* = 9	Postoperative sensitivity and tooth vitality	5	56%
		Fracture of material and retention	3	33%
		Tooth integrity	1	11%

**Table 6:** The overall FDI-2 criteria scores obtained at 5 years for the different categories

*n* = 95	EQUIA Forte						Tetric EvoCeram					
	Clinically excellent		Clinically good		Clinically satisfactory		Clinically excellent		Clinically good		Clinically satisfactory	
Aesthetic	66	69%	26	27%	3	3%	51	54%	40	42%	4	4%
Surface staining	78	82%	16	17%	1	1%	71	75%	21	22%	3	3%
Marginal staining*	72	76%	21	22%	2	2%	57	60%	34	36%	4	4%
Functional	52	55%	24	25%	19	20%	55	58%	28	29%	12	13%
Fracture of material and retention	78	82%	8	8%	9	9%	86	91%	5	5%	4	4%
Marginal adaptation	74	78%	20	21%	1	1%	65	68%	27	28%	3	3%
Approximal anatomic form – contact point	80	84%	1	1%	14	15%	86	91%	6	6%	3	3%
Patient’s view	81	85%	10	11%	4	4%	87	92%	5	5%	3	3%
Approximal anatomic form –contour	84	88%	6	6%	5	5%	91	96%	3	3%	1	1%
Biological	94	99%	0	0%	1	1%	95	100%	0	0%	0	0%
Postoperative sensitivity and tooth vitality	95	100%	0	0%	0	0%	95	100%	0	0%	0	0%
Recurrence of caries, erosion and abfraction	95	100%	0	0%	0	0%	95	100%	0	0%	0	0%
Tooth integrity	94	99%	0	0%	1	1%	95	100%	0	0%	0	0%
Periodontal response	95	100%	0	0%	0	0%	95	100%	0	0%	0	0%
Adjacent mucosa	95	100%	0	0%	0	0%	95	100%	0	0%	0	0%
Oral and general health	95	100%	0	0%	0	0%	95	100%	0	0%	0	0%

*Statistically significant at *p* < 0.05

For patients with both restorations scored as successful (FDI scores 1–3) in year 5 (*n* = 95), no statistically significant differences were found between EQUIA Forte and Tetric EvoCeram for functional and biological property scores (*p* > 0.05). A statistically significant difference, however, was found for the marginal staining: EQUIA Forte had a better result overall, with more restorations scored as ‘clinically excellent’, while the resin CO restorations were more frequently scored as ‘clinically good’ (*p* < 0.05; Fig. 4 and Table 6). To explore this observation even further, the comparison was made at each time point throughout the 5-year follow-up period. Statistically significant differences for marginal staining between the two investigated materials were found at every follow-up (*p* < 0.05).

## Discussion

As pointed out by the American Dental Association, a restorative material intended for use in posterior teeth needs to have a retention rate of at least 90% after 18 months of clinical service to become fully accepted as a definitive restorative material [32]. This clinical study found that the GH material, EQUIA Forte, was an acceptable restorative material with a retention rate of 94.5% after 5 years according to this criterion and that its survival and success rates were comparable to the nano-hybrid resin CO Tetric EvoCeram in two-surface class II restorations of posterior teeth.

These results are similar to those of a recent 5-year clinical study evaluating three high-viscosity glass-ionomer restorative materials in small class II cavities, with the main finding that the clinical results of the GH were comparable to those of resin CO [33]. However, a higher success rate was obtained for both EQUIA Forte and resin CO restorations, probably due to the smaller size of the cavities [33]. In a similar study design, it was found that the retention rate of EQUIA Forte after 2 years in extended class II cavities was relatively high (93.7%) and similar to the resin CO tested [25].

On the other hand, one clinical study found that after 1- and 2-year recall periods, the survival rate for EQUIA Forte restorations was significantly lower than that of the two resin CO materials used in layers or filled in bulk and recommended caution in the clinical use of that material [27, 34]. Although there are several differences in study design and methods, different evaluation criteria and possible sampling differences, the study compared independent samples for the two materials and likely had higher inter-patient variability. It is also possible that the difference between the materials was increased by using chlorhexidine as a disinfectant before the placement of restorative materials. The residual chlorhexidine might have interacted with phosphate in dentin due to the cationic part of the chlorhexidine molecule, which binds to negatively charged phosphate in dentin and, therefore, may have potentially impaired the bonding ability of GIC-based materials to tooth structures [35].

During the 5 years of this study, a comparable number of restorations made from EQUIA Forte and Tetric EvoCeram required replacement, with 8 and 9 replacements, respectively. The main reasons for replacements were post-operative sensitivity and material fractures. Similar results were reported by Heck et al. [20], with material fracture as the main reason for failure and restoration replacement for EQUIA Fil and Fuji IX GP Fast. The results of the present study are also supported by the findings of a meta-analysis by Beck et al. [14], which identified material fracture as the predominant type of failure in resin CO materials during the first 4 years of replacement. The number of EQUIA Forte restorations needing repair was higher, although not significantly different; there were 17 vs. 5 restoration repairs for EQUIA Forte and Tetric EvoCeram, respectively. Again, material fracture was the predominant reason for failure for both EQUIA Forte (100% of repairs) and Tetric EvoCeram (60% of repairs). In a systematic review, Ruengrungsom et al. [36] stated that marginal ridge chipping is a significant concern for two-surface GIC restorations, which is in accordance with the results of the current study, as repair due to ridge chipping of the material was predominantly observed in the EQUIA Forte group. Similar findings have been reported in two different clinical studies investigating the performance of EQUIA Fil in two-surface restorations [16, 18]. However, it must be noted that this study is not designed to analyse different reasons for failure, as the sample size for failed restorations is too small, and the data obtained can only be considered as a hypothesis for further research.

The evaluations of the restorations considered successful in the 5-year period revealed no statistically significant differences for the tested functional and biological FDI-2 properties. All functional properties showed deterioration over the 5-year study period. Regarding material fracture and retention, contact point and contour Tetric EvoCeram restorations showed somewhat better clinical performance, as CO restorations were scored as excellent in more than 90% of the cases at the last recall.

Interestingly, biological properties were scored as excellent for both materials throughout the study for all successful restorations, which did not require repair or replacement. Statistically significant differences were found for marginal staining between EQUIA Forte and Tetric EvoCeram (with proportions of ‘clinically excellent’ restorations of 76% and 60%, respectively). These are probably related to the known shrinkage of the resin CO material, leading to compromised marginal adaptation. A total of 78% of EQUIA Forte restorations had clinically excellent marginal adaptation, compared to 68% for Tetric EvoCeram; however, the difference was not statistically significant. The study did not evaluate other aesthetic properties because its primary objective was focused on assessing functional and biological properties. Furthermore, the colour and translucency characteristics of these two types of materials are fundamentally different, and the difference in the material’s appearance is obvious. Glass-hybrid is not available in as many shades as composite resin materials, its composition and structure are different, and it is rougher and cannot be polished with polishing rubbers, pastes, etc., so the colour match, surface gloss, lustre and translucency are not comparable to composite resin materials. In the posterior region, the aesthetic appearance of the restoration is not as important as in the anterior region. It does not mean that the aesthetic component in the posterior region should be disregarded but only that materials which are aesthetic, like glass-hybrids, although not as highly aesthetic as composite resin materials, can be used for the posterior restorations and that these are preferable to restorative materials which are not aesthetic, and which do not bond to hard dental tissues.

Several self-adhesive dental materials, such as, Activa Bioactive, Cention N, self-adhesive bulk-fill restorative and SureFil One, are also indicated as direct restorative options. However, they still have not gained the popularity of standard composite resins among clinicians for direct restorations. Results of the present study cannot be directly compared to the studies investigating self-adhesive restorative materials, because only short-term clinical performances of new materials are currently known. While the self-adhesive materials are applied in a bulk similarly to the glass hybrid material used in the present study, it is important to note that EQUIA Forte is a glass-ionomer-based material, while the others comprise modification with composite resin, i.e. alkasite, self-adhesive composite and ionic resin. van Dijken et al. [37] reported a failure rate of 24.1% after 1 year for Activa Bioactive and concluded that it must be used with an adhesive system. Oz et al. [38] compared Cention N (CN) to a resin composite in class II cavities. After 1 year, 3 CN restorations were lost, and 7 (18%) showed marginal adaptation problems. The survival rate for CN was 92.5%. Surefil One without adhesive was placed in different types of cavities and reviewed by dental practitioners in the USA [39]. After 1 year, one class II restoration was lost, and the colour match changed in 88% of the restorations. Nevertheless, the material was found to have clinically acceptable results. Cieplik et al. [40] compared the 1-year performance of a novel self-adhesive bulk-fill restorative (SABF) and a regular bulk-fill composite resin in class II restorations. They concluded that both materials were clinically acceptable according to FDI criteria. However, SABF had less surface lustre, colour match, translucency and marginal staining.

Although both materials yielded good clinical results, there were some limitations in the data analysis. In studies with a longer duration, it is expected that all patients and restorations included in the study cannot be re-examined for various reasons. The overall recall rate for the 5-year period was 73.9%, which is in accordance with prior power analysis, but it is still a limitation of the study. Scientific data confirm that the loss of patients for follow-ups in clinical studies is a general problem [14, 41]. Longer evaluation periods tend to result in higher dropout rates. A review of prospective studies with a 10-year evaluation time showed that the dropout of participants was high in 50% of the studies, with a mean dropout of 47% [42]. In addition, during the follow-up period of this study, the COVID-19 pandemic occurred in 2020, which affected the restoration follow-ups in every country. However, some of the patients reappeared at the fifth-year recall, but they had already received interventions from other dentists on the restorations or teeth included in the study, potentially impacting the failure rate. Moreover, some restorations were damaged during the operative procedures performed on the neighbouring tooth or, in some cases, due to prosthodontic or periodontal reasons.

Another limitation concerned the blinding of the operators and the evaluators as it was not possible to blind them to the materials because the differences were visually apparent, and the application procedures were distinct. This could introduce a potential risk of evaluator bias.

The generalisability of the study may also be impacted because it was conducted in university clinic settings. For this reason, the results obtained in clinical practice outside of universities could differ somewhat. However, since the GH material is less challenging to place, the risks of technical errors by the operator during restorative procedures are reduced. It would, therefore, be reasonable to assume that the potential bias due to the university setting of the study would not actually favour GH and that, in general clinical practice, the differences between the materials would likely be even less noticeable. However, further *in vivo* studies with longer observational periods would help obtain more data on the longevity of the tested materials.

In terms of success and survival rates, both the glass-hybrid restorative system and the nano-hybrid resin composite have demonstrated satisfactory performance and can be used as long-term restorative materials in the posterior region for moderate to large two-surface restorations.

## References

[1] Moraschini V, Fai CK, Alto RM, Dos Santos GO (2015). Amalgam and resin composite longevity of posterior restorations: a systematic review and meta-analysis. J Dent.

[2] Fisher J, Varenne B, Narvaez D, Vickers C (2018). The Minamata Convention and the phase down of dental amalgam. Bull World Health Organ.

[3] Frankenberger R, Dudek MC, Winter J, Braun A, Krämer N, von Stein-Lausnitz M, Roggendorf MJ (2020). Amalgam alternatives critically evaluated: effect of long-term thermos-mechanical loading on marginal quality, wear, and fracture behavior. J Adhes Dent.

[4] Minamata Convention on Mercury(n.d.). Overview. https://mercuryconvention.org/en/parties/overview (accessed 14 July 2023).

[5] Mylonas P, Zhang J, Banerjee A (2021). Conventional glass-ionomer cements; a guide for practitioners. Dent Update.

[6] Opdam NJ, Bronkhorst EM, Loomans BA, Huysmans MC (2010) 12-year survival of composite vs. amalgam restorations. J Dent Res 89:1063-1067. 10.1177/0022034510376071 10.1177/0022034510376071 20660797

[7] Sidhu SK (2011). Glass-ionomer cement restorative materials: a sticky subject?. Aust Dent.

[8] Moshaverinia A, Roohpaour N, WWL C, Schricker SR (2011). A review of powdermodifications in conventional glass-ionomer dental cements. J Mater Chem.

[9] Zhang J, Braun P, Banerjee A (2020). In vitro compressive strength and edge stability testing of directly repaired glass-ionomer cements. Clin Oral Investig.

[10] Spagnuolo G (2022). Bioactive dental materials: the current status. Materials (Basel).

[11] Vetromilla BM, Opdam NJ, Leida FL, Sarkis-Onofre R, Demarco FF, van der Loo MPJ, Cenci MS, Pereira-Cenci T (2020). Treatment options for large posterior restorations: a systematic reviewand network meta-analysis. J Am Dent Assoc.

[12] Friedl K, Hiller KA, Friedl KH (2011). Clinical performance of a new glass ionomer based restoration system: a retrospective cohort study. Dent Mater.

[13] Basso M, Brambilla E, Benite MG, Giovannardi M, Ionescu AC (2015). Glass ionomer cement for permanent dental restorations: a 48-months, multi-centre, prospective clinical trial. Stoma Edu J.

[14] Beck F, Lettner S, Graf A, Bitriol B, Dumitrescu N, Bauer P, Moritz A, Schedle A (2015). Survival of direct resin restorations in posterior teeth within a 19-year period (1996-2015): a meta-analysis of prospective studies. Dent Mater.

[15] Gurgan S, Kutuk ZB, Ergin E, Oztas SS, Cakir FY (2015). Four-year randomized clinical trialto evaluate theclinical performance of a glass ionomer restorative system. Oper Dent.

[16] Klinke T, Daboul A, Turek A, Frankenberger R, Hickel R, Biffar R (2016). Clinical performance during 48 months of two current glass ionomer restorative systems with coatings: a randomized clinical trial in the field. Trials..

[17] Turkun LS, Kanik O (2016). A prospective six-year clinical study evaluating reinforced glassionomer cements with resin coating on posterior teeth: Quo Vadis?. Oper Dent.

[18] Fotiadou C, Frasheri I, Reymus M, Diegritz C, Kessler A, Manhart J, Hickel R, Klinke T, Heck K (2019). A 3-year controlled randomized clinical study on the performance of two glass-ionomer cements in class II cavities of permanent teeth. Quintessence Int.

[19] Gurgan S, Kutuk ZB, Cakir FY, Ergin E (2020) A randomized controlled 10 years follow up of a glass ionomer restorative material in class I and class II cavities. J Dent 94:103175. 10.1016/j.jdent.2019.07.013 10.1016/j.jdent.2019.07.013 31351909

[20] Heck K, Frasheri I, Diegritz C, Manhart J, Hickel R, Fotiadou C (2020). Six-year results of a randomized controlled clinical trial of two glass ionomer cements in class II cavities. J Dent.

[21] Zhang OL, Niu JY, Yin IX, Yu OY, Mei ML, Chu CH (2023). Bioactive materials for caries management: a literature review. Dent J (Basel)..

[22] Moshaverinia M, Navas A, Jahedmanesh N, Shah KC, Moshaverinia A, Ansari S (2019). Comparative evaluation of the physical properties of a reinforced glass ionomer dental restorative material. J Prosthet Dent.

[23] Brkanovic S, Ivanisevic A, Miletic I, Mezdic D, Jukic Krmek S (2021). Effect of nano-filled protective coating and different pH environment on wear resistance of new glass hybrid restorative material. Materials(Basel).

[24] Miletic I, Baraba A, Basso M, Pulcini MG, Markovic D, Peric T, Atalayin C, Turkun LS (2020). Clinical performance of a glass-hybrid system compared with a resin composite in the posterior region: results of a 2-year multi-centre study. J Adhes Dent.

[25] Gurgan S, Kutuk ZB, Ozturk C, Soleimani R, Cakir FY (2020). Clinical performance of a glass-hybrid restorative in extended class II cavities. Oper Dent.

[26] Grossi JA, Cabral RN, APD R, Leal SC (2018). Glass hybrid restorations as an alternative for restoring hypomineralized molars in the ART model. BMC Oral Health.

[27] Balkaya H, Arslan S (2020). A two-year clinical comparison of three different restorative materials inclass II cavities. Oper Dent.

[28] Schwendicke F, Frencken JE, Bjørndal L, Maltz M, Manton DJ, Ricketts D, Van Landuyt K, Banerjee A, Campus G, Doméjean S, Fontana M, Leal S, Lo E, Machiulskiene V, Schulte A, Splieth C, Zandona AF, Innes NP (2020). Managing carious lesions: consensus recommendations on carious tissue removal. Adv Dent Res.

[29] Hickel R, Peschke A, Tyas M, Mjor I, Bayne S, Peters M, Hiller KA, Randall R, Vanherle G, Heintze SD (2010). FDI World Dental Federation - clinical criteria for the evaluation of direct and indirect restorations. Update and clinical examples. J Adhes Dent.

[30] Hickel R, Peschke A, Tyas M, Mjor I, Bayne S, Peters M, Hiller KA, Randall R, Vanherle G, Heintze SD (2010). FDI World Dental Federation - clinical criteria for the evaluation of direct and indirect restorations - update and clinical examples. Clin Oral Invest.

[31] Hickel R, Mesinger S, Opdam N, Loomans B, Frankenberger R, Cadenaro M, Burgess J, Peschke A, Heintze SD, Kühnisch J (2023). Revised FDI criteria for evaluating direct and indirect dental restorations - recommendations for its clinical use, interpretation, and reporting. Clin Oral Invest.

[32] ADA Council on Scientific Affairs (1998). ADA council on dental benefit programs. Statement on posterior resin-base composites. J Am Dent Assoc.

[33] Wafaie RA, Ibahim Ali A, SAE E-N, Mahmoud SH (2023). Five-year randomized clinical trial to evaluate the clinical performance of high-viscosity glass ionomer restorative systems in small class II restorations. J Esthet Restor Dent.

[34] Balkaya H, Arslan S, Pala K (2019). A randomized, prospective clinical study evaluating effectiveness of a bulk-fill composite resin, a conventional composite resin and a reinforced glass ionomer in class II cavities: One-year results. J Appl Oral Sci.

[35] Borompiyasawat P, Putraphan B, Luangworakhun S, Sukarawan W, Techatharatip O (2022). Chlorhexidine gluconate enhances the remineralization effect of high viscosity glass ionomer cementon dentin carious lesions in vitro. BMC Oral Health.

[36] Ruengrungsom C, JEA P, Burrow MF (2018). Comparison of ART and conventional techniques on clinical performance of glass-ionomer cement restorations in load bearing areas of permanent and primary dentitions: a systematic review. J Dent.

[37] van Dijken JWV, Pallesen U, Benetti A (2019). A randomized controlled evaluation of posterior resin restorations of an altered resin modified glass-ionomer cement with claimed bioactivity. Dent Mater.

[38] Oz FD, Meral E, Gurgan S (2023). Clinical performance of an alkasite-based bioactive restorative in class II cavities: a randomized clinical trial. J Appl Oral Sci.

[39] Rathke A, Pfefferkorn F, McGuire MK, Heard RH, Seemann R (2022). One-year clinical results of restorations using a novel self-adhesive resin-based bulk-fill restorative. Sci Rep.

[40] Cieplik F, Scholz KJ, Anthony JC, Tabenski I, Ettenberger S, Hiller KA, Buchalla W, Federlin M (2022). One-year results of a novel self-adhesive bulk-fill restorative and a conventional bulk-fill composite in class II cavities-a randomized clinical split-mouth study. Clin Oral Investig.

[41] von Gehren MO, Rüttermann S, Romanos GE, Herrmann E, Gerhardt-Szép S (2023). A 23-year observational follow-up clinical evaluation of direct posterior composite restorations. Dent J.

[42] van Dijken JW, Pallesen U (2014). A randomized 10-year prospective follow-up of class II nano-hybrid and conventional hybrid resin composite restorations. J Adhes Dent.

